# Three-dimensional seismic response analysis of connected structures with damping layers

**DOI:** 10.1371/journal.pone.0353116

**Published:** 2026-07-10

**Authors:** Chenghao Xu, Dewen Liu, Kangjie Ling, Xiaopeng Li

**Affiliations:** 1 School of Civil Engineering & Architecture, Wenzhou Polytechnic, Wenzhou, Zhejiang, China; 2 International College, Krirk University, Bangkok, Thailand; 3 College of Civil Engineering, Southwest Forestry University, Kunming, Yunnan, China; Universiti Teknologi Malaysia, MALAYSIA

## Abstract

To enhance the seismic damping effect of connected structures under intense seismic activities, this study introduces a novel control system for connected structures. This system strategically places the damping layers at different locations within the main towers to mitigate the violent system response of the connected structure during rare earthquakes. The double-sided damping structure mainly involves arranging damping layers in the towers on both sides of the connected structure. The damping layers use lead-core isolation bearings (LRB), which fully combines the concepts of damping and isolation. It applies isolation technology to the damping system of high-rise structures, and can effectively solve the technical problem of overturning when isolation technology is applied to high-rise buildings in engineering experiments. The structure is modeled using ABAQUS. Models for both single-sided and double-sided damping structures are established and subsequently compared with the seismic resistance structure. This comparison is conducted to analyze the time-history response of layer shear, base reaction force, top-layer acceleration, and interstory drift of various structural models under rare earthquakes. Additionally, shear-drift hysteresis curves of the damping layer are constructed to evaluate the energy dissipation rate of the structure’ s damping layer using an energy analysis method. The results show that both damping structures exhibit superior energy dissipation and damping effects. However, the introduction of an additional damping layer in the double-sided damping structure results in a more obvious damping effect and a higher energy dissipation rate. The risk of overturning due to excessive bending deformation of the superstructure is mitigated by designing the location of the damping layer. However, the control effect of both damping structures on vertical earthquakes is not obvious, and further research is needed.

## Introduction

Connected structure connects two or more buildings through a connecting corridor (The sky corridor of a connected structure serves to connect two towers) forming a unique structural shape and bringing a strong visual impact. In recent years, this type of structure has been increasingly used in real projects and has become a new landmark in many cities [1]. Although the towers of connected structures can be effectively connected to each other, the existence of connecting corridors complicates the stress situation under the influence of earthquakes [2]. Therefore, the structural design problems of such buildings are receiving increasing attention. This type of building has become a focus of attention for designers and researchers [3–4].

There are various connection forms of connecting corridors, which can be generally categorized into strong and weak connections. The connecting corridor connection form [5] and connection location [6] have a great influence on the damage characteristics of the connected structure, the damage form, and the response mode of the dynamics. Among them, the weak connection connection weakens the connection relationship between the connecting corridor and the towers, and the deformation capacity between the towers is relatively independent, which largely reduces the irregularity of the main structure [7], and the force at the connecting corridor and the connection bearing is also simpler, so it can also be taken to design the vibration damping measures such as the setting of dampers at the connection or the seismic isolation bearing [8–9]. Therefore, many designers and researchers prefer to set the structure into the form of weak connection to mitigate the effect of earthquake on the connected structure, but it is worth noting that the deformation of the two sides of the tower is relatively independent, and the relative drift is too large under the action of strong earthquakes, the connecting corridor is prone to collide with the tower or fall off, and the superstructure has the risk of overall collapse [10]. When the connecting corridor employs rigid connection, the main body of multiple structures coordinates the deformation，This leads to a significant impact on the force situation of the entire structure due to the connecting corridor [11–12].The towers influence each other, a strong connection can easily result in severe damage to the connecting corridors and supports due to an increase in its own stiffness, thereby affecting the safety of the structure [13]. Especially this is extremely serious when the elevated connecting corridors are strongly connected to the main towers [14]. To address these problems, Chen et al. [15] installed dampers inside the towers and at the connection supports. Wang Xiaonan et al. [16] analyzed the vibration reduction of connecting corridors that were connected by a strong connection, by installing a flexural restraint brace at the connection. However, the relative vibration reduction effect was found to be somewhat limited. Their research only adds dampers locally in the corridor or tower, without considering the inter-story isolation layer as an overall control strategy.

**Fig 1:**
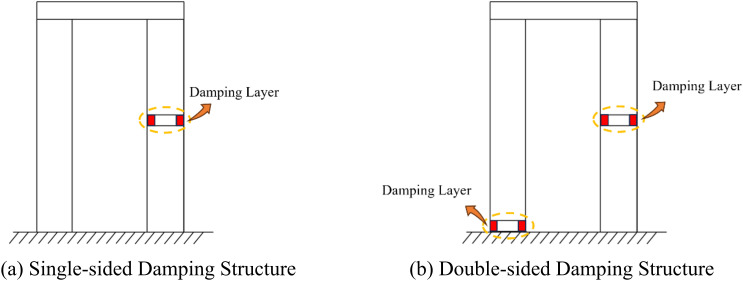
Schematic diagram of the damping system.

Liu Liangkun et al. [17] proposed a novel structural control system for connected structures. This system incorporates a damping layer (Floors in a building structure designed to absorb seismic energy)in one tower, as depicted in Fig 1(a). The system establishes a damping layer on only one side, effectively preventing the upper structure from overturning due to excessive drift. Simultaneously, it dissipates a portion of the earthquake energy through the damping layer, thereby achieving energy dissipation and damping effects. However, this study only installed damping layers in the single-sided tower and did not consider or solve the problem that single-sided control is detrimental to the other side of the tower. Lin et al. [18] studied that multi-dimensional and multi-point earthquakes, together with the traveling wave effect, significantly amplify the responses of long-span connected structures, and that seismic design must simultaneously consider the multi-dimensional components and traveling wave excitations with apparent velocity differences. Lyu et al. [19] constructed a shared tuned mass damper (STMD) system for two towers with flexible connections and a corridor, derived a three-degree-of-freedom analytical model, revealed its shock absorption mechanism, established an optimization parameter formula aiming at minimizing the displacements of the two towers, and verified its significant vibration reduction and control effects through numerical simulation using the El Centro seismic wave. Zheng et al. [20] proposed a built-in vibration reduction layer system for rigidly connected connected buildings; after simplifying and optimizing the model, the vibration energy was reduced by 69% and the response by 30–50%, with the optimal arrangement at the low-rise levels. Tao et al. [21] focused on the steel truss-RC shear wall joints in connected structures and compared two types of truss-wall connection structures: ordinary and friction energy-dissipating ones. The friction-type joints showed an increase of 34.9% in ultimate displacement and 123% in cumulative energy dissipation, confirming that truss-wall joints can efficiently transmit seismic forces. Wei et al. [22] constructed a soil-connected structure coupling model, revealing that ground fissure sites significantly amplify the translational-torsional response and advance the hysteretic peak; sliding bearings can suppress the translational-torsional coupling under strong earthquakes, and the inter-story drift angle still meets the specifications. Liang et al. [23] found that cushioned pile-raft foundations can significantly reduce the transmission of seismic motion to the superstructure by allowing a certain degree of sliding between the piles and the raft, thereby effectively lowering the seismic response of the structure. This demonstrates clear seismic isolation and vibration reduction advantages, especially under strong earthquake loading. Zhang et al. [24] proposed the TNSAD to control the isolated corridor of high-rise connected structures, reducing the displacement of the isolation layer by 20–40% and the acceleration of the corridor by 30–40%, with stable effects under multiple seismic conditions, and the energy dissipation increased to six times that of viscous dampers. Existing studies on connected structures have covered vibration reduction technologies for rigid/weak connections, truss-wall joints, complex sites, and high-rise isolated corridors, but there have been no systematic research results on the Double-sided Damping Structure. Liang et al. [25] showed that cushion-isolated foundations possess favorable seismic isolation and energy dissipation capacity. The shear characteristics of the cushion–structure interface directly affect the isolation performance, and a rational combination of cushion materials and layer thickness configuration can further enhance the seismic reduction performance of the foundation. Yang et al. [26] demonstrated that the disconnected pile-raft foundation (DPRF), by introducing a cushion layer between the piles and the raft, can effectively provide seismic isolation, reduce the transmission of earthquake effects to the superstructure and piles, and exhibit greater vibration reduction potential than the traditional connected pile-raft foundation. Relevant studies have shown that seismic isolation structures have significant energy dissipation advantages.

Building on the research of previous scholars, this paper introduces a double-sided damper structure, as depicted in Fig 1(b) In this structure, the top connects two towers on both sides through a rigid connecting corridor. The damping layer is located at the base of the left tower and the middle of the right tower. When traditional base isolation structures are applied to structures with a large height-to-width ratio, the risk of structural overturning increases due to excessive bending deformation, leading to tensile forces in the base isolation bearing of the isolation layer [27]. However, the double-sided damping structure mitigates the risk of superstructure overturning caused by the application of isolation technology at the base location by integrating the energy dissipation method of the damping layer. For the tower on the right-hand side, the risk of overturning is significantly reduced. This is because the superstructure of the inter-layer damping layer can be effectively controlled due to the installation of the damping layer only in the middle. Moreover, the structure can utilize the relative drift between the two substructures after the installation of the damping layer to consume energy, which fully combines the concepts of damping and isolation. It applies isolation technology to the damping system of high-rise structures, and can effectively solve the technical problem of overturning when isolation technology is applied to high-rise buildings in engineering experiments. The damping layer offers a wide range of energy-consuming devices to choose from, including high damping rubber bearings, lead rubber bearings, and viscous dampers. This paper is mainly to lead rubber bearing design vibration damping layer for research.

## Analysis of the particle model and establishment of the finite element model

### Analysis of the particle model

**Fig 2:**
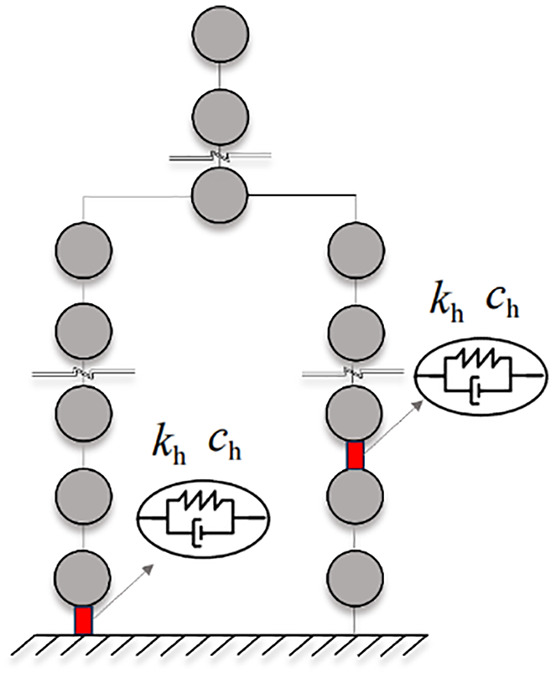
Particle model.

At present, the mainstream simplified models for connected structures include the continuous model, series rigid body model, and lumped mass model. This study focuses on the horizontal dynamic characteristics of the structure, thus adopting the lumped mass model as the simplification method. For simplification, several basic assumptions are made as follows: 1) The mass of each floor is lumped at the corresponding floor slab; 2) Only horizontal vibration is considered; 3) The left and right towers as well as the rigid connecting part are dominated by shear deformation; 4) Axial deformation of the left and right towers and the connecting part is neglected. Finally, the simplified model is obtained as shown in Fig 2, where the stiffness of the seismic isolation layer is $k_{\text{h}}$ and the damping is $c_{\text{h}}$.

According to D’Alembert’s principle, the dynamic equation of the double-sided seismic isolation lumped mass model can be expressed as:


$$\begin{array}{c} M\ddot{x}+C\dot{x}+Kx=-MIx_{g} \end{array}$$ (1)

$x=[X_{L};X_{C};X_{R}]$, $X_{L};X_{C};X_{R}$ represent the drift responses of the left tower, connecting corridor floor, and right tower, with similar expressions applicable to their acceleration and velocity responses，I is a unit vector，$x_{g}$ is earthquake response acceleration.

The mass matrix in the formula can be expressed as:$\left[\begin{array}{ccc} M_{L} & \text{0} & \text{0}\\ \text{0} & M_{C} & \text{0}\\ \text{0} & \text{0} & M_{R} \end{array}\right]$; The stiffness matrix of the model can be expressed as $\left[\begin{array}{ccc} K_{L}+k_{h} & \text{0} & \text{0}\\ \text{0} & K_{C} & \text{0}\\ \text{0} & \text{0} & K_{R}+k_{h} \end{array}\right]$; The damping matrix of the overall model is$\left[\begin{array}{ccc} C_{L}+c_{h} & \text{0} & \text{0}\\ \text{0} & C_{C} & \text{0}\\ \text{0} & \text{0} & C_{R}+c_{h} \end{array}\right]$.

Compared with the traditional seismic resistance model, the double-sided seismic isolation model achieves an effect similar to that of architectural seismic damping systems by introducing corresponding damping and adjusting the stiffness at the position of the seismic isolation layer. Meanwhile, since the seismic isolation layer introduced in the left tower is located at the bottom, this is analogous to traditional base isolation structures.

### Project overview

**Fig 3:**
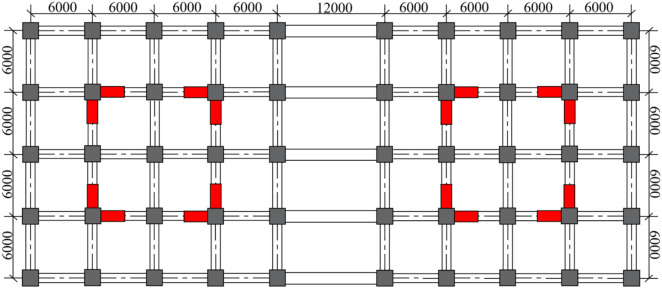
Layout plan of structures.

A certain high-rise double-tower connected frame shear wall structure is designed with a seismic protection class of B. It has a seismic protection intensity of 8 degrees and a basic seismic acceleration of 0.2g. The design is grouped under the second seismic category, and the site category is Class II. The structure comprises 18 floors, each with a height of 3.6m and a floor thickness of 120 mm, culminating in a total structure height of 64.8m. The connecting corridor, located at the top of the structure, spans 12m and consists of 4 floors. The layout plan of the structure is illustrated in Fig 3. The structural rebar employs HRB400, the concrete grade for the beams and columns is C40, and the concrete grade for the floor slab is C30. The elastic modulus of concrete is 32500 MPa, and the elastic modulus of steel bars is 200 GPa. The floor dead load is 3kN/m2, and the floor live load is 2kN/m2. The self-weight of the infill wall and other loads are uniformly distributed on the frame beam, resulting in a uniform line load of 8kN/m. The connecting corridors utilize a curtain wall as the peripheral partition, with a uniform line load of 1.8kN/m. The beam and column cross-section parameters of the structure are presented in Table 1.

**Table 1:** The section parameters of beams and columns.

Component type	Floor	Section dimension (mm × mm)
Framework column	1-18	800 × 800
Framework beam	1-18	350 × 700
Connecting corridor beam	14-18	400 × 800
Coupling beam	1-18	350 × 650
Embedded beam	1-18	350 × 500

### Modeling information

In this study, numerical models were established using the general-purpose finite element software ABAQUS. The beams and columns were modeled based on the concept of fiber beam elements, where the cross-sections were divided into layers to distribute integration points. By increasing the density of integration points at the positions of steel reinforcement, the influence of steel on concrete was incorporated into the model. The fiber beam elements were defined using the B31 element type. For the floor slabs and core structures, a layered shell concept was adopted, in which the shell elements were divided into layers with unequal thicknesses, and the mechanical behaviors of each layer were defined separately to simulate the role of steel reinforcement. The shell element type used in this study was S4R.

The beams and columns were modeled using B31 fiber beam elements, and the floor slabs and core-wall components were modeled using S4R layered shell elements. The approximate mesh size was 60 mm for the beam-column members and 60 mm for the shell components. A mesh sensitivity analysis was conducted by comparing models with mesh sizes of 180 mm, 60 mm, and 30 mm. The differences in the key response indices, including top-story acceleration and interstory drift, were within 5%; therefore, the medium mesh was adopted in the subsequent analyses.

**Fig 4:**
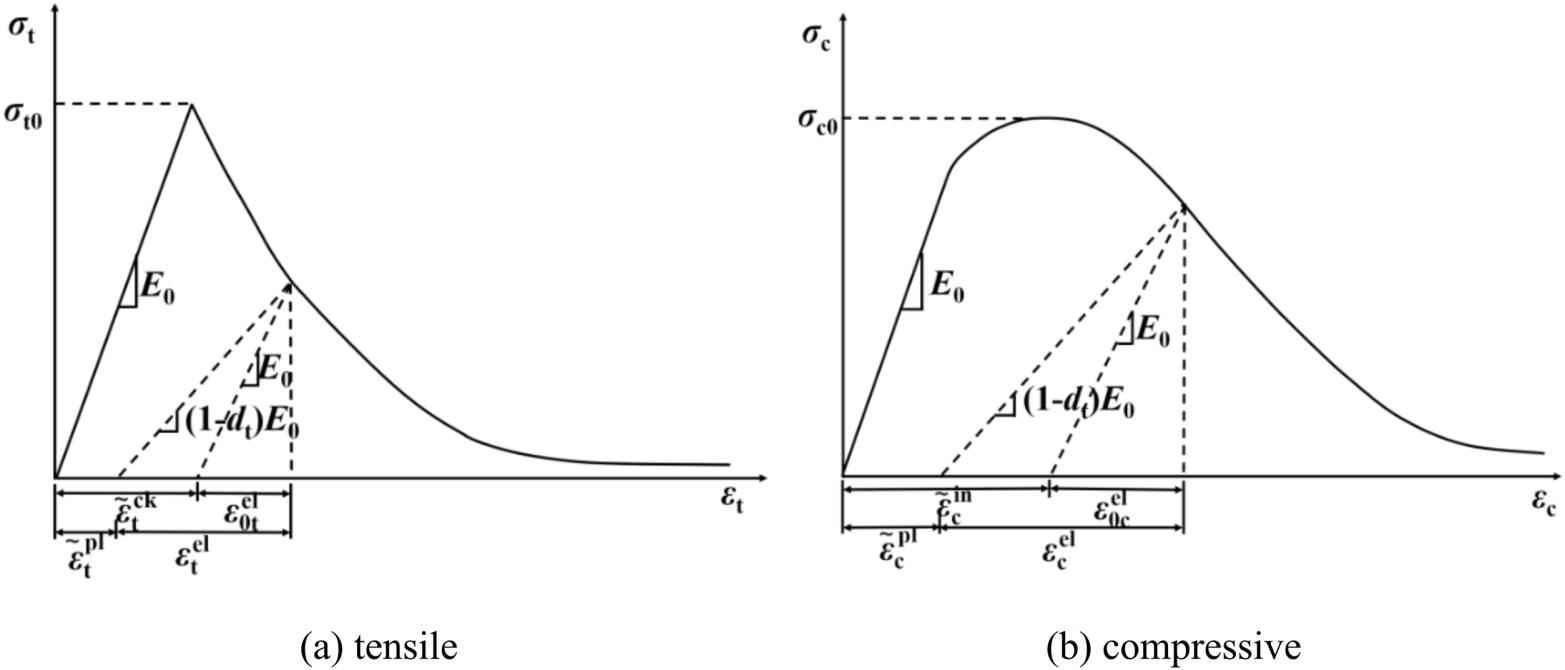
Schematic diagrams of uniaxial tensile-compressive damage and stiffness recovery.

Due to the highly nonlinear mechanical behavior of concrete, simplifications were necessary for the simulation. The constitutive behavior of concrete was defined using the built-in Concrete Damaged Plasticity (CDP) model in ABAQUS, with its stress-strain curve input into the software. A schematic diagram of this stress-strain curve is shown in the Fig 4. The main CDP parameters were set as follows: dilation angle = 35°, eccentricity = 0.1, *f*_*b0*_*/* *f*_*c0*_ = 1.16, *Kc* = 0.667, and viscosity parameter = 0.0005.

In contrast, steel reinforcement, whose mechanical properties are approximately isotropic, was modeled using a bilinear elasto-plastic model. Its mechanical behavior was defined by specifying the initial stiffness $E_{s}$, yield stiffness 0.01 $E_{s}$, and yield force $F_{y}$.

**Fig 5:**
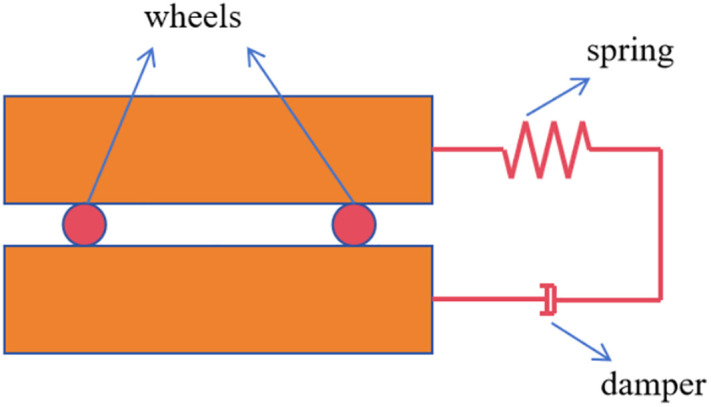
Simplified model of bearing.

The lead-rubber isolation bearing consumes energy through the deformation of the bearing itself, and it can also change the stiffness of the structure, prolong the natural period of the structure, improve the response mode of structure, and reduce the risk of failure of the structure under the action of large earthquakes, and the simplified model of the Lead-Core Rubber Isolation Bearing is shown in Fig 5. Therefore, in this paper, the Lead-Core Rubber Isolation Bearing is selected as the energy-consuming device of the structure to establish the damping model.

The control equation for lead-rubber isolation bearings are established based on the forced vibration regime for a single-mass system [28]:


$$\begin{array}{c} m\ddot{u}+c\dot{u}+ku=F \end{array}$$ (2)

* m is the equivalent mass of the isolation bearing, $\ddot{u}$ is the acceleration of the isolation bearing, c is the equivalent damping coefficient of the seismic isolation bearing, $\dot{u}$ is the velocity of the isolation bearing, k is the equivalent stiffness of the seismic isolation bearing, u is the drift of the seismic isolation bearing, F is the horizontal reaction force of the isolation bearing.

Due to the small mass of the isolation bearing itself, the inertia force generated is much smaller than the restoring force and damping force of the bearing, and the control equations of the isolation bearing can be further simplified as follows:


$$\begin{array}{c} \dot{u}+ku=F \end{array}$$ (3)

When the elastic-plastic time history analysis of the structure is carried out in the software, due to the strong nonlinearity of the structure, in order to calculate efficiently, this paper simulates the damping of the isolation bearing by introducing the plastic deformation of the bearing [29], and simplifies the mechanical behaviors of the isolation bearing through the linear hardening model.

### The damping scheme design and structural modeling

**Fig 6:**
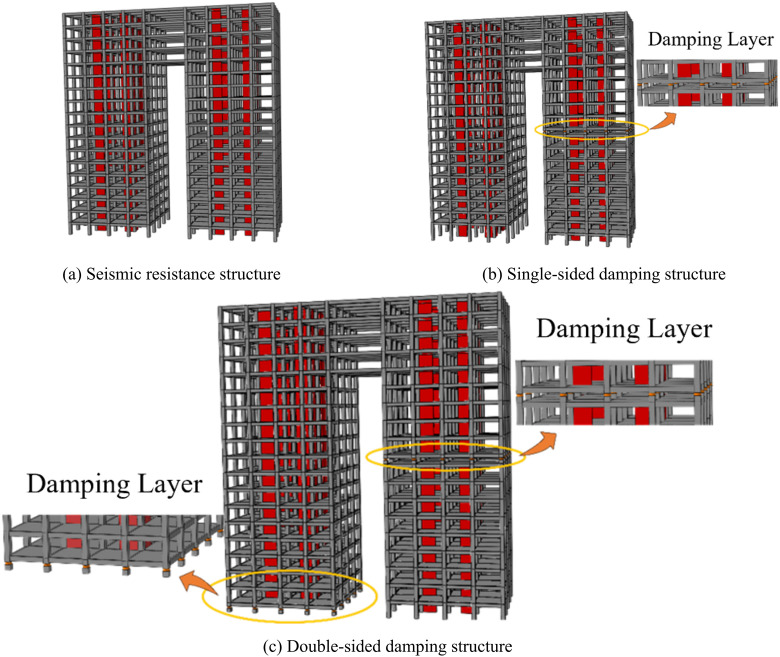
3D model of structures.

In this study, the damping layer is positioned on the ninth floor of the right tower, following the recommendations of GB/T 50011-2010 [30]. for the placement of the seismic isolation layer. A single-sided damping structure was initially established through the design and modeling of the damping model. Subsequently, a double-sided was established by introducing an additional damping layer at the base of the left tower. Concurrently, to investigate the damping effect of the two damping models, a seismic model was established for comparison. Each structural model is depicted in Fig 6.

**Fig 7:**
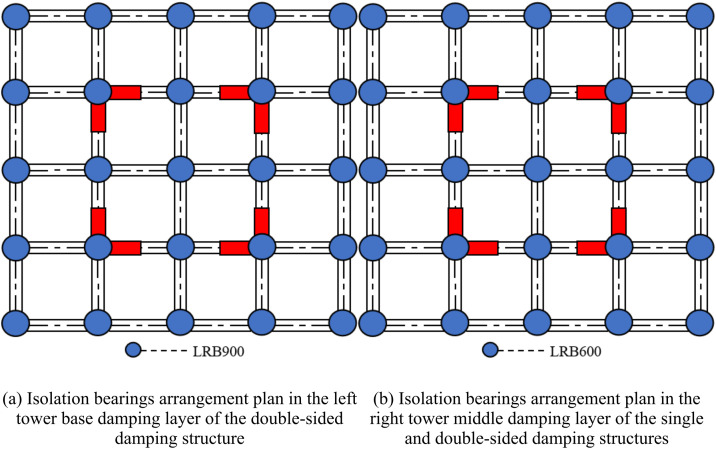
Isolation bearings arrangement plan of the single and double-sided damping structures.

Per the “GB/T 50011-2010”, which stipulates the face pressure limit value for selecting the number and type of isolation bearings, the bottom damping layer of the left tower utilizes LRB900, and the seismic isolation layer of the right tower employs LRB600. The bearing arrangement diagram is depicted in Fig 7, and the parameters of the isolation bearings are presented in Table 2.

**Table 2:** Performance parameters of lead-rubber bearings.

Bearing type	Effective diameter/mm	Vertical stiffness $K_{V}$/(KN/mm)	Equivalent horizontal stiffness $K_{eq}$/(KN/mm)	Stiffness before yield $K_{u}$/(KN/mm)	Yield force $Q_{d}$/KN	Rubber thickness t/mm
LRB900	900	2480	2.41	17.24	211	180
LRB600	600	1581	1.58	11.57	91.4	120

### Modal analysis

The first six natural periods of the structures are presented in Table 3. The natural periods of both damping structures have been effectively extended. The introduction of an additional damping layer at the base location further extends the natural periods of the double-sided damping structures, which makes the natural periods of the structure more effective in avoiding the predominant period of the site and reducing the response of the structure under the seismic action

**Table 3:** Natural periods of the structures.

Order	Seismic resistance structure (s)	Single-sided damping structure (s)	Double-sided damping structure (s)
1	1.54	2.85	3.35
2	1.53	2.11	3.29
3	1.45	1.71	3.08
4	0.46	0.59	0.91
5	0.45	0.59	0.88
6	0.45	0.58	0.81

#### Material Rayleigh damping.

In this paper, the Rayleigh damping is used to define the material damping of the structure. The Rayleigh damping assumes that the damping force of the structure has a linear relationship with the mass and stiffness of the structure to construct the mechanical model. Its mathematical expression is as follow:


[C]=α[M]+β[K] (4)

* [C] is the damping matrix，[M] is the mass matrix，[K] is the stiffness matrix of structures，α and β are the mass damping coefficient and the stiffness damping coefficient*

The mass damping coefficient α and the stiffness damping coefficient β are calculated according to the following two formulas:


$$\begin{array}{c} \alpha =\frac{\text{2}\omega_{i}\omega_{j}\left(\xi_{i}\omega_{j}-\xi_{j}\omega_{i}\right)}{{\omega_{j}}^{\text{2}}-{\omega_{i}}^{\text{2}}} \end{array}$$ (5)

$$\begin{array}{c} \beta =\frac{\text{2}\left(\xi_{i}\omega_{j}-\xi_{j}\omega_{i}\right)}{{\omega_{j}}^{\text{2}}-{\omega_{i}}^{\text{2}}} \end{array}$$ (6)

* $\omega_{i},\omega_{j}$ are the frequency values corresponding to the two periods used for calculating the damping coefficient, and $\xi_{i},\;\xi_{j}$ are the damping ratios of the corresponding periods.

The Rayleigh damping coefficients α and β were calculated based on the modal analysis results. The damping ratios of the reinforcing steel and concrete were taken as 2% and 5%, respectively.

## Time history analysis of structures

**Fig 8:**
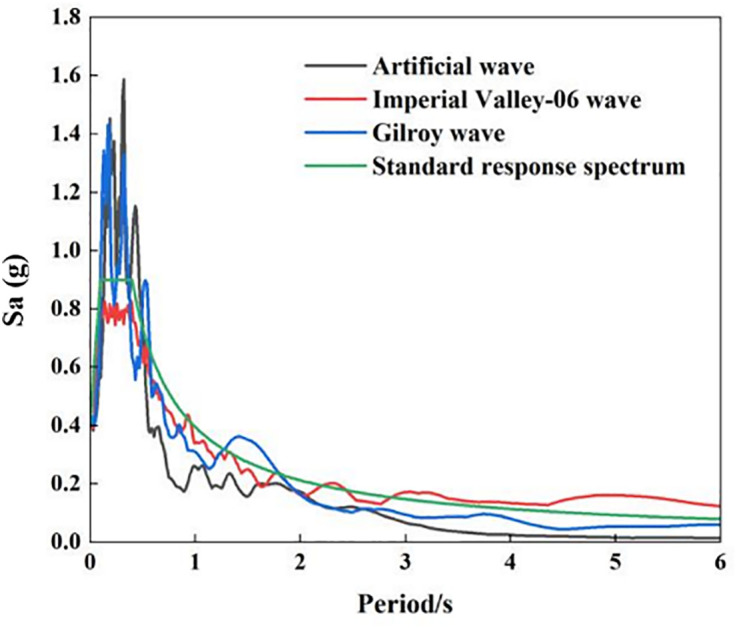
Acceleration response spectrum.

When conducting time-history analysis, it is necessary to select appropriate seismic waves to make the calculation results more representative. This study selects waves based on site conditions. The equivalent shear wave velocity of the soil layer at the site is between 250 m/s and 500 m/s. In accordance with the requirements of “GB/T 50011-2010”, and refer to ASCE/SEI 7-22 [31], 3 seismic waves are selected, including two natural waves and one artificial wave. According to the code requirements, the response values corresponding to the first four natural vibration periods of the structure should not deviate from the standard response spectrum by more than 20%. In this paper, the first four periods of the three structures are taken as characteristic points for wave selection. Two natural waves that meet the main period of the building and the dominant period of the building site are selected from multiple ground motion records collected by the Pacific Earthquake Engineering Research Center in the United States, and one artificial wave that meets the requirements is randomly generated. The basic information of the selected ground motion records is shown in Table 4, and the acceleration response spectra are shown in Fig 8.

**Table 4:** Basic information of ground seismic records.

Event	Time	Tg/g	Earthquake magnitude	Station
ArtWave	/	0.40	/	/
Imperial Valley-06	1979	0.36	6.53	USGS STATION 5053
Gilroy	1989	0.33	4.9	WEATHER STATION

According to the seismic design requirements, the peak accelerations of the three selected seismic waves were scaled to 0.4g for the elastoplastic time-history analysis under rare earthquakes with a seismic intensity of 8 degrees. The peak acceleration inputs in the X, Y, and Z directions were scaled with a ratio of 1:0.85:0.65. To quantitatively compare the seismic response control effect of different structural models, the response reduction rate is defined as follows:


$$\begin{array}{c} R=\frac{S_{n}\;-S_{i}}{S_{n}}\times \text{100}\% \end{array}$$

where R is the response reduction rate, $S_{n}$ is the seismic response value of the seismic-resistant structure, and $S_{i}$ is the seismic response value of the damping structure. A positive value of R indicates that the seismic response of the damping structure is reduced compared with that of the seismic-resistant structure.

### Interstory drift of structures

**Fig 9:**
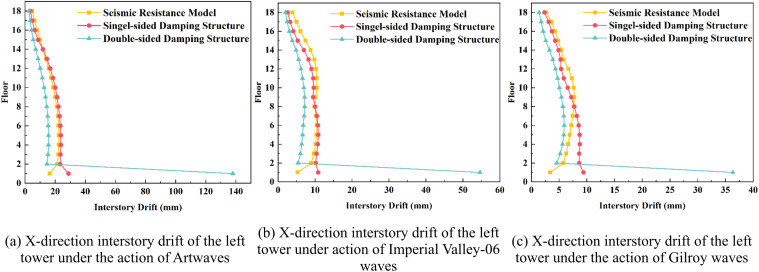
X-direction interstory drift of the left tower of each structure under the action of seismic waves.

**Fig 10:**
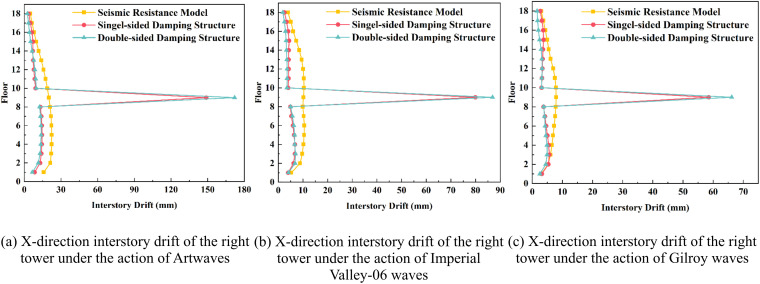
X-direction interstory drift of the right tower of each structure under the action of seismic waves.

**Fig 11:**
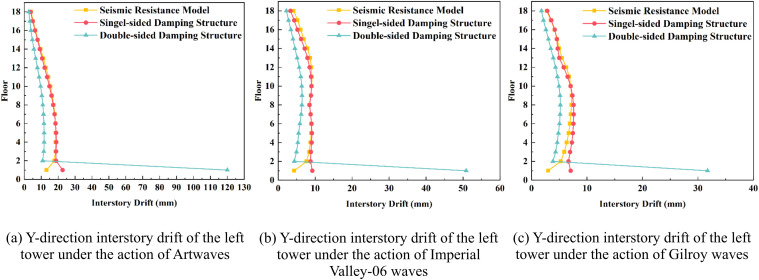
Y-direction interstory drift of the left tower of each structure under the action of seismic waves.

**Fig 12:**
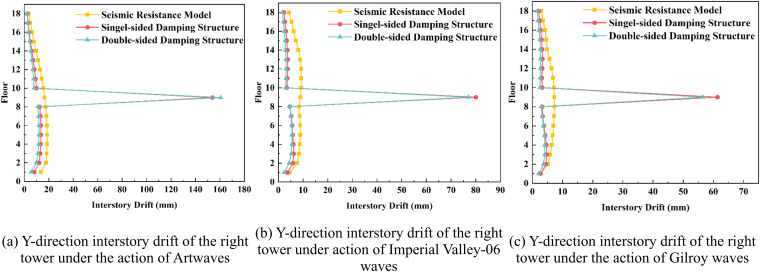
Y-direction interstory drift of the right tower of each structure under the action of seismic waves.

By inputting artificial wave, Imperial Valley-06 wave and Gilroy wave for the time-history analysis of seismic resistance structure, single-sided damping structure, and double-sided damping structure respectively, the interstory drift of each model under seismic action are obtained, as shown in Figs 9–12. Both the single-sided and double-sided damping structures introduce the damping layer, and the stiffness of the damping layer is relatively small, and the deformation of the structure is mainly concentrated in the damping layer, and the interstory drift of the damping layer of the structure is the largest. Maximum interstory drift of the damping layer is 148 mm, which is smaller than the limit drift of the isolation bearing required by the “Code for seismic design of buildings”, which is less than 330 mm.

Under rare earthquakes, the interstory drift angles of the structures do not exceed the elastic-plastic interstory drift angle limit of 1/100. Among them, the interstory drift angle of the double-sided damping structure is significantly reduced due to the introduction of the base damping layer in the left tower. However, for the left tower in the single-sided damping structure, the inter-story drift did not show a significant improvement compared with that of the seismic-resistant structure, and even increased in some stories. This is mainly because the introduction of the vibration-control layer in the right tower reduced and redistributed the overall structural stiffness, thereby altering the vibration characteristics of the whole structure and affecting the displacement response of the left tower. As shown in the figure, this increase is mainly concentrated in the 1st to 9th stories of the left tower, while the displacement of the 10th to 18th stories is effectively controlled. This indicates that although the vibration-control layer contributes to displacement control in the upper stories, the reduction in overall stiffness and the change in mode shape also aggravate the displacement response in the lower stories of the left tower.

### Base reaction force and interlayer shear force

Under the action of rare earthquakes with a seismic intensity of 8 degrees, the base reaction forces of the three structural models under the selected seismic waves are listed in Table 5. Compared with the seismic-resistant structure, the horizontal base reaction forces of both damping structures are reduced to different degrees. In the X direction, the base reaction force of the single-sided damping structure is reduced by 2.9%, 16.9%, and 3.0% under the ArtWave, Imperial Valley-06, and Gilroy waves, respectively. For the double-sided damping structure, the corresponding reductions are 21.9%, 32.7%, and 22.8%, respectively. This indicates that the additional damping layer at the base of the left tower further reduces the horizontal base reaction force and improves the overall seismic response control effect. By contrast, the vertical base reaction forces of the three structures show no obvious reduction. This is because the selected LRB-based damping layers mainly provide horizontal energy dissipation capacity, while their vertical energy dissipation capacity is limited, which is consistent with the findings of Liu et al. (2021) [17].

**Table 5:** Base reaction force of structures.

Structure type	Event	X direction（KN）	Y- direction（KN）	Z- direction（KN）
Seismic resistance structure	Artwaves	31122	27341	440218
	Imperial Valley-06 Waves	27618	23723	476052
	GilroyWaves	24429	21670	491268
Single-sided damping structure	Artwaves	30219	26830	441577
	Imperial Valley-06 Waves	22961	20885	463712
	Gilroy Waves	23694	19989	486351
Double-sided damping structure	Artwaves	24310	22210	445823
	Imperial Valley-06 Waves	18574	16565	463690
	Gilroy Waves	18860	16576	488740

**Fig 13:**
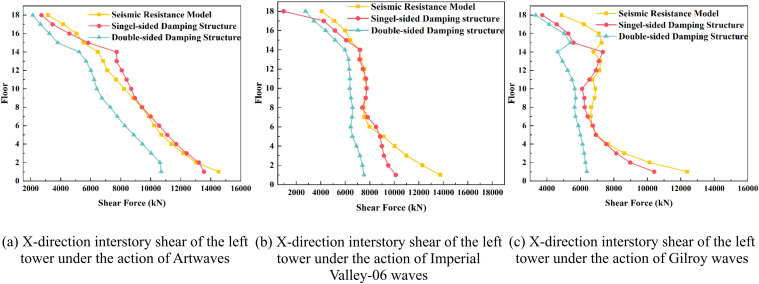
X-direction interstory shear of the left tower of each structure under the action of seismic waves.

**Fig 14:**
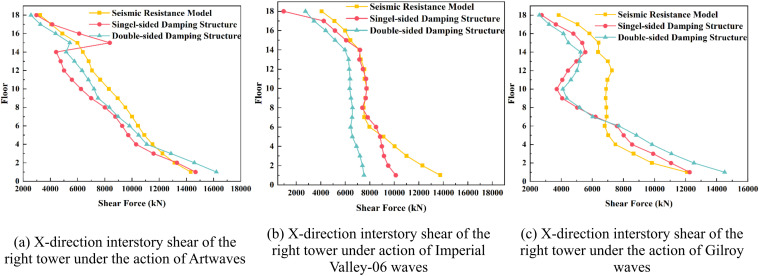
X-direction interstory shear of the right tower of each structure under the action of seismic waves.

**Fig 15:**
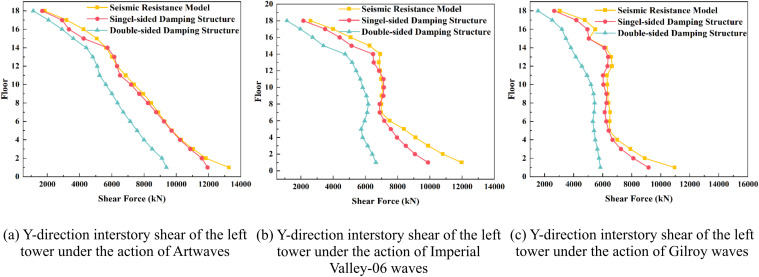
Y-direction interstory shear of the left tower of each structure under the action of seismic waves.

**Fig 16:**
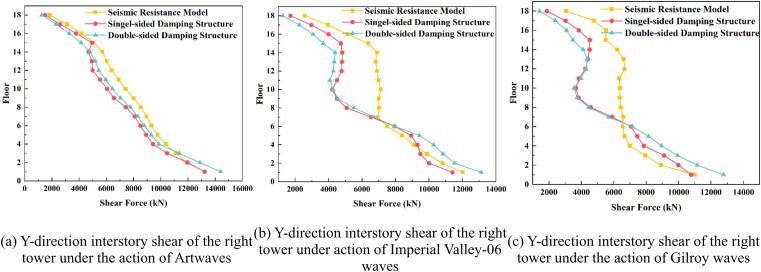
Y-direction interstory shear of the right tower of each structure under the action of seismic waves.

Interstory shear of the structure is shown in Figs 13–16. It can be seen that the interstory shear of the left tower of the double-sided damping structure has a significant reduction relative to the other two structures, and the single-damping structure has a slight amplification relative to the seismic resistance structure, which is related to the damping layer of the single-damping structure is set up in the middle of the right tower, due to the damping layer is set up in the middle of the right tower, which leads to a significant reduction of the structure’s stiffness in the location of the damping layer, which is not conducive to the left tower’s seismic resistance, this corresponds to the case of interstory drift in section 3.1.

### Top-story acceleration of structures

Top-story acceleration of the two towers of the three structures is selected as the reference for comparison. The envelope value of top acceleration of the structure is shown in Table 6. As can be seen from the table, because the structure is connected by a rigid connecting corridor, the stiffness of the superstructure is larger, and there is no serious whiplash effect at the top of the seismic resistance structure. X-direction top acceleration envelope value of the double-sided seismic structure is less than the peak ground acceleration, which indicates that the structure has better seismic performance. However, the envelope values of the peak acceleration in the Y-direction for each structure are generally amplified relative to the peak ground acceleration. This amplification is attributed to the relatively reduced stiffness of the structure in Y-direction compared to X-direction.

**Table 6:** Top-story acceleration of structures.

Structure type	Event	X-direction of left tower （m/s2）	Y-direction of left tower （m/s2）	X-direction of right tower（m/s2）	Y-direction of right tower（m/s2）
Seismic resistance structure	Artwaves	3.84	3.4	3.86	3.38
	Imperial Valley-06 Waves	4.7	4.146	4.96	4.23
	Gilroy Waves	4.48	3.95	4.6	4.0
Single-sided damping structure	Artwaves	4.0	3.57	4.02	3.31
	Imperial Valley-06 Waves	4.23	3.8	4.28	3.6
	Gilroy Waves	4.48	3.89	4.59	3.74
Double-sided damping structure	Artwaves	3.4	3.06	3.41	2.88
	Imperial Valley-06 Waves	3.78	3.5	3.85	3.6
	Gilroy Waves	3.9	3.4	3.9	3.7

From the quantitative analysis results of the acceleration at the top of the structure, the double-sided damping structure exhibits the optimal vibration reduction control effect, while the single-sided damping structure also has a certain vibration reduction effect. Taking the seismic-resistant structure as the benchmark, under the action of three seismic waves: Artwaves, Imperial Valley-06 Waves, and Gilroy Waves, the accelerations in the X and Y directions of both the left and right towers of the double-sided damping structure are significantly reduced, with a reduction range of 7.50%−22.38% and an average reduction of approximately 14%. Among them, the X direction of the right tower has the largest reduction (22.38%) under Imperial Valley-06 Waves, showing a prominent vibration reduction advantage. In contrast, the single-sided damping structure only has a small reduction of 2%−15% in some directions under Imperial Valley-06 Waves and Gilroy Waves. However, under the action of Artwaves, the accelerations in the X/Y directions of the left tower and the X direction of the right tower are instead increased by 4%−5% compared with the seismic-resistant structure, failing to achieve effective vibration reduction under all working conditions. In addition, the differences in acceleration values between the left and right towers of the three structure types are all ≤0.2 m/s², and the vibration reduction trends of the two towers are consistent, with no obvious tower eccentricity effect. This further confirms the stability and superiority of the double-sided damping structure in controlling the top acceleration.

### Isolation bearing reaction force

**Fig 17:**
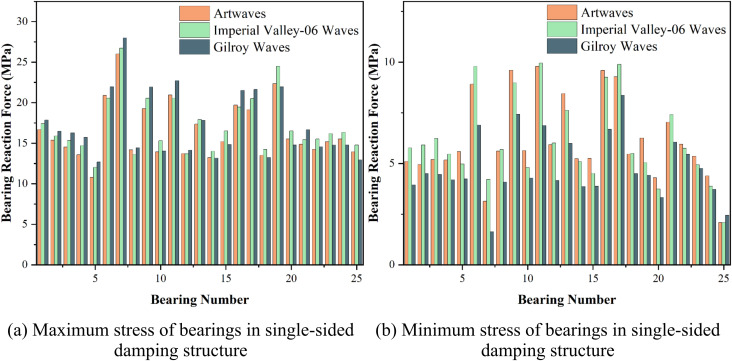
Stress of bearings in single-sided damping structure.

**Fig 18:**
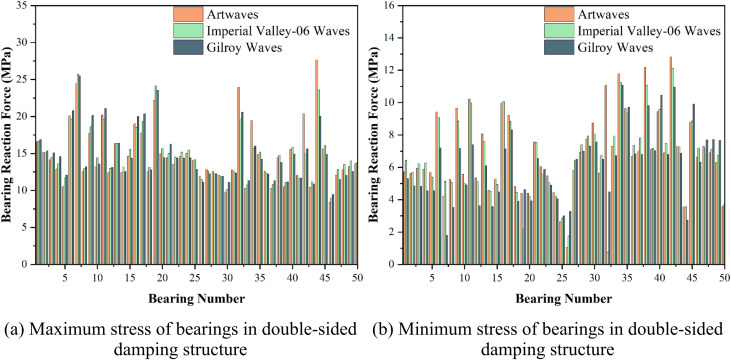
Stress of bearings in double-sided damping structure.

Bearing reaction force is an important index to reflect the stability of the structure under strong seismic action. If the tensile stress or compressive stress of the bearing exceeds the license, there is a risk of overall overturning of the superstructure. According to the ‘Code for Seismic Design of Buildings’ [9], the provisions for isolation bearings stipulate that during rare earthquakes, the tensile stress on the seismic isolation bearing should not exceed 1 MPa, and the compressive stress should not surpass 30 MPa. The reaction force of the bearing, as depicted in Figs 17–18, remains in a pressurized state under seismic action. Maximum compressive stress is 27 MPa, and Minimum compressive stress is 0.7 MPa. These values do not exceed the relevant standards, indicating no risk of the superstructure overturning. This validates that the design of the damping layer within the seismic structure is sound. The structure enhances the safety redundancy of the conventional base isolation structure against overturning, achieved through a reasonable combination of damping layers.

## Analysis of the energy method

The control equation for a multi-degree of freedom forced vibration system under the action of an earthquake are:


$$\left[M]\left\{\ddot{U}(t)\right\}+[C]\left\{\dot{U}(t)\right\}+\left[F(t)\right]=-[M]{\ddot{U}}_{g}(t\right)$$ (7)

* [M],  [C] are the mass and damping of the structural system, respectively, [F(t)] are the restoring force column vectors of the structural system, and $\dot{U}(t),\;\dot{U}(t),\;{\ddot{U}}_{g}(t)$ are the relative velocity, relative acceleration and ground motion acceleration of the structural system, respectively.

Processing the control equation for a multi-degree of freedom forced vibration system under the action of an earthquake,according to the principle of work done by force and drift, by multiplying the drift of a single calculation step on both sides of the equilibrium equation and integrating over time, that is, letting, $\left\{U\right\}=\left\{\dot{U}\right\}dt$, the accumulation process of energy can be obtained:


$$\begin{array}{c} \int_{\text{0}}^{t}{\left\{\dot{U}\right\}}^{T}[M]\left[\dot{U}\right]dt+\int_{\text{0}}^{t}{\left\{\dot{U}\right\}}^{T}[C]\left[\dot{U}\right]dt+\int_{\text{0}}^{t}{\left\{\dot{U}\right\}}^{T}[F]\left[\dot{U}\right]dt=-\int_{\text{0}}^{t}{\left\{\dot{U}\right\}}^{T}[M]\left[{\ddot{U}}_{g}\right]dt \end{array}$$ (8)

The input energy equation is:


$$\begin{array}{c} E_{i}(t)=\int_{\text{0}}^{t}{\left\{\dot{U}\right\}}^{T}[M]\left[{\ddot{U}}_{g}\right]dt \end{array}$$ (9)

And the energy dissipation of the bearing can be taken as $E_{z}(t)=\int_{\text{0}}^{t}F_{z}(u)du$ for calculation, where $F_{z}(u)$ is the horizontal reaction force of the damping layer and u is the inter-layer drift of the damping layer, u=u(t).

### Energy dissipation capacity of the damping layer

**Fig 19:**
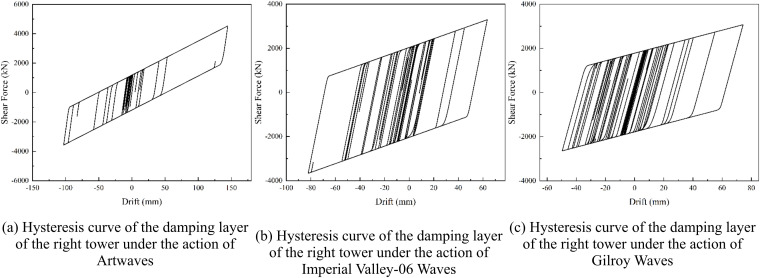
Hysteresis curve of the damping layer in the single-sided damping structure.

**Fig 20:**
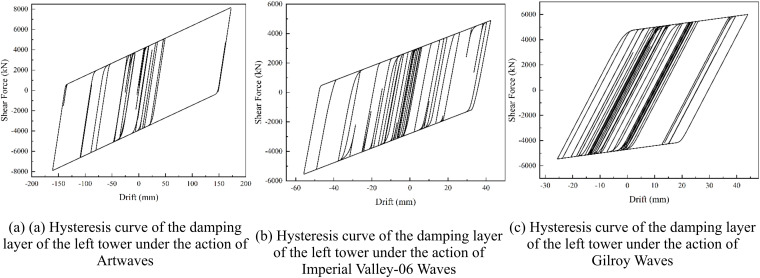
Hysteresis curve of the damping layer of the left tower of the double-sided damping structure.

**Fig 21:**
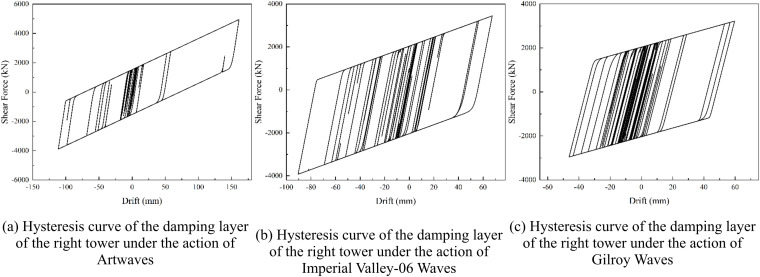
Hysteresis curve of the damping layer of the right tower of the double-sided damping structure.

LRBs reduce the seismic response of structures by absorbing or consuming the seismic energy input through large plastic deformation. In this paper, the horizontal shear curve *V(t)* and the corresponding drift curve *S(t)* of the damping layer of the structure are used to construct the energy dissipation hysteresis curve of the damping layer, which visually reacts to the energy dissipation capacity of the damping layer. Taking X-direction as an example, the shear-drift hysteresis curves of the damping layer are shown in figures. From Figs 19–21, it can be seen that the energy dissipation hysteresis curves of the damping layer of the structure are full, which indicates that the damping structure has an excellent energy dissipation and damping effect, and the double-sided damping structure has an even better energy dissipation effect due to the fact that one of the damping layers is placed at the base of left tower, which results in greater drift and shear force in the damping layer, leading to more excellent energy dissipation effects.

Since the two damping layers of the structure are located at different positions, their stiffness is not completely consistent. When the structure is subjected to external excitation, the two damping layers will interact with each other, which will cause the damping bearing to be constrained within one cycle. In addition, in order to better simulate the role of the lead core when defining the LRB, the damping coefficient of the bearing is defined, so that the mechanical properties of the bearing are related to the speed to a certain extent, which leads to the fact that the displacement and the load are not completely positively correlated [32].

### Energy dissipation rate of the damping layer

In order to accurately study the energy dissipation of the isolation bearing, the energy dissipation rate parameter of the isolation bearing is introduced. The comparison of the energy dissipation and damping layers of the two damping structures is shown in Table 7.

**Table 7:** Energy of Structures.

Structure Category	seismic waves	Input energy (kN·m)	Energy dissipation of the damping layer (kN·m)	Energy dissipation rate (%)	Average energy dissipation rate (%)
Single-sided damping structure	Artwaves	34812	4005	11.5	10.7
	Imperial Valley-06 Waves	31093	2996	9.6	
	Gilroy Waves	29095	3271	11.2	
Double-sided damping structure	Artwaves	38905	9949	25.5	23.56
	Imperial Valley-06 Waves	30621	7336	23.9	
	Gilroy Waves	28478	6058	21.2	

As can be seen from Table 7, under the action of three seismic waves, the energy dissipation rates of the single-sided damping structure are 11.5%, 9.6%, and 11.2%, with an average energy dissipation rate of 10.7%. In contrast, the energy dissipation rates of the double-sided damping structure are 25.5%, 23.9%, and 21.2%, with an average energy dissipation rate of approximately 23.6%. The energy dissipation rate of the double-sided damping structure, which uses two damping layers, has significantly improved compared to the single-sided damping structure with only one damping layer. This is mainly because the deformations on both sides of the double-sided damping structure are coordinated, and both damping layers of the structure participate in energy dissipation. This indicates that the double-sided damping structure has higher safety redundancy under the action of rare earthquake, and has superior damping performance.

## Discussion

Based on previous studies, this research constructs a double-sided damping structure. The model introduces damping layers at different positions of two different towers through isolation technology, forming a damping scheme for high-rise connected buildings. This scheme effectively solves the horizontal shock absorption problem of high-rise connected structures, and the overall horizontal acceleration, interstory shear, and interstory drift of the structure are effectively controlled.

However, the existing research scheme still has shortcomings. Since the right tower adopts the form of interstory isolation to construct the damping layer, this will lead to poor shock absorption effect on certain floors [32]. In subsequent studies, research can be conducted by adding certain viscous dampers to the corresponding floors. In addition, the vertical shock absorption of the structure is basically ineffective. The vertical stiffness of the LRB90 bearing is 2480 kN/mm, while the horizontal equivalent shear stiffness is 2.41 kN/mm, with a difference of more than a thousand times between the two. The excessively large vertical stiffness results in small deformation in the vertical direction, and there is no effective energy-dissipating device in the vertical direction, which is extremely unfavorable for the vertical shock absorption of the structure. In subsequent studies, the vertical shock absorption performance can be improved by introducing disc springs that can change the vertical stiffness.

To further advance the structure into practical engineering applications, further substructure experiments are needed to verify the structural response under actual boundary conditions. The damping layer also needs to add certain limiting devices to improve the safety redundancy of the structure.

## Conclusion

In this paper, a control system of connected structure with energy damping layer at different locations of two towers is proposed, the response of the structure under rare seismic action is analyzed, and the energy dissipation capacity of different damping structure is analyzed by energy method, and the main conclusions are as follows:

[1] The introduction of LRBs to construct the damping layer can extend the natural period of the structure and reduce the response of the structure under seismic action.
[2] The introduction of damping layer in the middle of one side only can reduce the seismic response of the structure to a certain extent, which is mainly reflected in the tower with added damping layer. However, the introduction of the damping layer on one side only leads to a sudden change in the stiffness of the structure at a local location, which is not conducive to the seismic performance of the tower on the side without a damping layer, and there is no advantage in the interstory drift and interstory shear of the tower on the side without a damping layer relative to the conventional seismic resistance structure. This indicates that, in practical design, the stiffness redistribution caused by local damping-layer arrangement should be carefully considered to avoid adverse effects on the seismic performance of the adjacent tower.
[3] The introduction of a damping layer at the base of one tower and at the center of the other tower can significantly reduce the seismic response of the structure. The value of interstory drift, story shear, and energy dissipation capacity of the structure are greatly improved compared to traditional seismic structures, and the safety redundancy of the structure can be greatly increased. These results suggest that this arrangement has good application potential in engineering practice, as it can provide a more favorable balance between response control and structural safety under rare earthquakes.
[4] The connected structure with energy-consuming damping layer added on both sides effectively avoids the tensile situation of the bottom isolation bearing due to the bending of the building structure that may occur in the traditional application of base isolation. Simultaneously, the deformation coordination ability of the twin towers significantly reduces the risk of excessive drift in the isolation layer of traditional interstory isolation structures, which could lead to the overall overturning of the superstructure of the isolation layer. Therefore, the proposed system can provide useful reference for improving design strategies of connected high-rise buildings, especially in enhancing anti-overturning capacity and ensuring deformation compatibility of the twin towers.
[5] The two damping structures have excellent damping effects in the horizontal direction, but there is no significant improvement in the damping performance in the vertical direction, and the damping capacity in the vertical direction needs further study. From the perspective of practical engineering decision-making, the proposed damping system may increase the initial construction cost to some extent, but its advantages in reducing seismic damage, repair demand, and potential collapse risk indicate considerable life-cycle economic benefits.

Overall, the results show that the rational arrangement of energy-dissipating damping layers can effectively improve the seismic performance, safety redundancy, and collapse-resistance capacity of connected structures. These findings can provide a reference for engineering design, design guidelines, and cost-benefit evaluation of connected high-rise buildings in seismic regions.
